# Biomarkers of oxidative stress in saliva in pigs: analytical validation and changes in lactation

**DOI:** 10.1186/s12917-019-1875-z

**Published:** 2019-05-14

**Authors:** Camila Peres Rubio, Eva Mainau, José Joaquín Cerón, Maria Dolores Contreras-Aguilar, Silvia Martínez-Subiela, Elena Navarro, Fernando Tecles, Xavier Manteca, Damian Escribano

**Affiliations:** 1 Interdisciplinary Laboratory of Clinical Analysis Interlab-UMU, Faculty of Veterinary Medicine, Regional Campus of International Excellence ‘Campus Mare Nostrum’, University of Murcia, Espinardo, Murcia, 30100 Spain; 2 Department of Animal and Food Science, School of Veterinary Science, Universitat Autònoma de Barcelona, 08193 Bellaterra, Barcelona Spain

**Keywords:** Antioxidant, Oxidant, Reactive oxygen species, Saliva, Sows

## Abstract

**Background:**

Biomarkers of oxidative stress in pigs have been measured in serum/plasma samples. However, blood collection in pigs can be highly stressful to the animals. Saliva is a biological fluid with several advantages in pigs over blood, since it can be easily collected without stress to the animals, being therefore an ideal sample in this species. The objective of this study was the validation of assays for the evaluation of oxidative stress status in saliva of pigs. For this purpose, three assays commonly used to evaluate the total antioxidant capacity (TAC): trolox equivalent antioxidant capacity (TEAC), cupric reducing antioxidant capacity (CUPRAC), and ferric reducing ability of plasma (FRAP)), one individual antioxidant (uric acid) and two assays to evaluate oxidant concentrations (advanced oxidation protein products (AOPP) and hydrogen peroxide (H_2_O_2_)) were measured and validated in porcine saliva. In addition, the possible changes of these assays in sows’ saliva during lactation were be studied.

**Results:**

The methods had intra- and inter-assays coefficient of variation lower than 15%. They also showed an adequate linearity and recovery, and their detection limits were low enough to detect the analytes in saliva of pigs. Overall the analytical validation tests showed that the assays used in our study are valid and reliable for the evaluation of oxidative stress in porcine saliva. In addition, it was observed that these salivary biomarkers can change in a situation of oxidative stress such as lactation in sows.

**Conclusions:**

All assays for salivary biomarkers of oxidative stress evaluated in this study have demonstrated a high analytical accuracy and low imprecision. In addition, it has been observed that these biomarkers showed significant changes in a situation of oxidative stress such as lactation in sows. Therefore, this study opens a new possibility of using saliva as a non-invasive sample to evaluate oxidative stress in pigs.

## Background

Oxidative stress is a term used to indicate an imbalance between the production of reactive oxygen species (ROS) in the organism and the ability of the antioxidant molecules to neutralize them [1]. ROS formation leads to the accumulation of proinflammatory substances, which further contributes to more generation of these oxidants [2]. A ROS accumulation results in oxidative damage to lipids, proteins and DNA and consequently tissue injury, therefore the oxidative stress is involved in pathogenesis of many diseases. In pigs, oxidative stress has been linked to several diseases and clinical conditions such as experimental endotoxemia or septic shock [3], respiratory infections [4], porcine reproductive and respiratory syndrome (PRSS) [5], and skin disease [6]. ROS accumulation can also induce damage in the intestinal tissue, which can facilitate bacterial translocation and compromise the intestinal barrier integrity in pigs [2].

In addition to diseases, changes in oxidative stress biomarkers in pigs can occur at different situations during their productive period which are associated with variations in the oxidant balance of the animals. For example, ROS increase during transportation, resulting in an oxidative stress [7–9]. The weaning period also has been linked with oxidative stress producing an increase of ROS [10]. In addition, the serum levels of ROS and thiobarbituric acid reactive substances (TBARS) are higher during gestation (days 90 and 109) and lactation (days 1 and 3) than in early gestation (day 10) [11].

In addition to the above mentioned biomarkers of oxidative stress, total antioxidant capacity (TAC) assays, such as the ferric reducing ability of plasma (FRAP) has also been measured in serum of pigs in studies mainly related with the evaluation of different dietary supplementations [12, 13]. Moreover, Trolox equivalent antioxidant capacity (TEAC), another TAC assay, has been used to evaluate TAC in plasma of piglets undergoing weaning-induced stress [14].

To the authors' knowledge, all these reports regarding biomarkers of oxidative stress in pigs have measured them in serum/plasma samples. However, blood collection in pigs can be highly stressful to the animals since it implies animal immobilization, and how this stress can affect the levels of the different oxidant biomarkers is unknown. Saliva is a biological fluid with several advantages in pigs over blood, since it can be easily collected without stress to the animals, being therefore an ideal sample in this species. Although biomarkers of oxidative stress have been widely measured in saliva in humans [15–17], to the authors’ knowledge they have not been previously measured in porcine saliva.

The objective of this study is the validation of assays for the evaluation of oxidative stress status in saliva of pigs. In addition, the possible changes in oxidative stress biomarkers in sows’ saliva during lactation, which is a period in which variations in oxidative stress are known, will be studied. For this purpose, three assays commonly used to evaluate the total antioxidant capacity (TAC) (TEAC, cupric reducing antioxidant capacity (CUPRAC), and FRAP), one individual antioxidant (uric acid) and two assays to evaluate oxidant concentrations (advanced oxidation protein products (AOPP) and hydrogen peroxide (H_2_O_2_)) will be included in this report.

## Results

### Analytical validation

Intra- and inter-assay coefficients of variation (CVs) were below 8 and 15%, respectively for all the assays evaluated (Table 1). The mean accuracy assessed by the recovery study in saliva samples was 90% for TEAC, 98% for CUPRAC, 89% for FRAP, 97% for uric acid, 70% for AOPP and 83% for H_2_O_2_. Linearity under dilution in saliva samples resulted in linear regression equations with correlation coefficients higher than 0.99 in all cases. The limit of detection (LOD) was 0.09 mmol/L for TEAC, 0.003 mmol/L for CUPRAC, 0.031 mmol/L for FRAP, 0.031 mg/dL for uric acid, 3.67 μmol/L for AOPP and 0.001 μmol/L for H_2_O_2_.

**Table 1:** Mean, standard deviation (SD) and intra- and inter-assay coefficients of variation (CVs) in each analyte concentrations of three saliva samples of pigs (A, low concentrations; B, medium concentrations; C, high concentrations)

	Intra-assay			Inter-assay		
	Mean	SD	CV (%)	Mean	SD	CV (%)
TEAC (mmol/L)						
A	0.06	0.00	6.32	0.06	0.01	10.76
B	0.15	0.00	2.40	0.14	0.01	4.35
C	0.31	0.01	1.78	0.30	0.01	3.56
CUPRAC (mmol/L)						
A	0.04	0.00	3.03	0.04	0.01	14.81
B	0.11	0.00	0.82	0.11	0.00	3.11
C	0.24	0.00	0.31	0.25	0.00	2.02
FRAP (mmol/L)						
A	0.09	0.01	6.37	0.09	0.00	4.25
B	0.23	0.00	1.43	0.23	0.01	3.17
C	0.40	0.02	5.11	0.40	0.01	3.28
Uric acid (mg/dL)						
A	0.11	0.01	4.80	0.09	0.01	12.59
B	0.54	0.01	2.27	0.52	0.01	2.63
C	1.65	0.01	0.79	1.65	0.02	1.25
AOPP (μmol/L)						
A	28.06	0.50	1.79	24.08	2.00	8.31
B	72.84	1.16	1.59	66.49	8.95	13.46
C	213.02	3.23	1.52	185.59	21.25	11.45
H_2_O_2_ (μmol/L)						
A	9.74	0.69	7.14	8.41	1.21	14.34
B	14.58	0.48	3.27	13.58	1.50	11.07
C	19.62	1.06	5.40	18.40	0.86	4.66

### Changes of the biomarkers in saliva during lactation

**Fig. 1:**
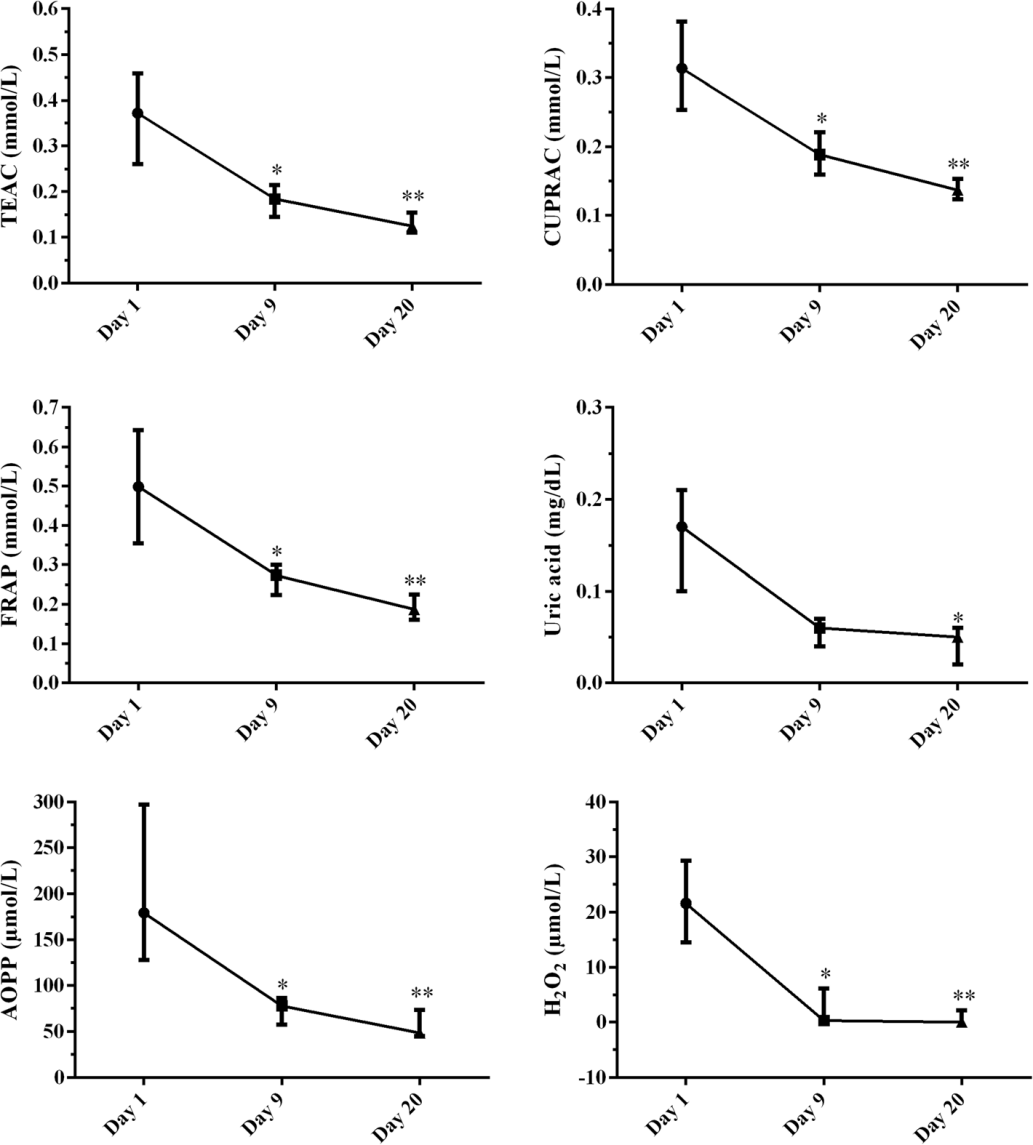
Results for saliva concentrations of trolox equivalent antioxidant capacity (TEAC), cupric reducing antioxidant capacity (CUPRAC), ferric reducing ability of plasma (FRAP), uric acid, advanced oxidation protein products (AOPP) and hydrogen peroxide (H_2_O_2_) in sows during the first, 9th and 20th day of lactation (median with interquartile range). Asterisks indicate significant differences between the first day of lactation. * *P* ≤ 0.01, ** *P* ≤ 0.0001

Figure 1 shows the antioxidant and oxidant concentrations in saliva of sows during lactation. All salivary biomarkers of oxidative stress analyzed in our study were higher at first day of lactation and then decreased during the following days of lactation. Salivary TEAC, CUPRAC, FRAP, AOPP and H_2_O_2_ were significantly lower at day 9 (*P* ≤ 0.01) and 20 (*P* ≤ 0.0001) than at day 1 of lactation. Salivary uric acid decreased significantly at day 20 (*P* ≤ 0.0001) of lactation compared to day 1.

The values of TEAC in saliva were 2.0 and 2.9-fold lower at 9 and 20 day of lactation, respectively, than those of the first day. Salivary CUPRAC and FRAP decreased 1.6 and 1.7-fold, respectively, at day 9, and 2.2 and 2.5-fold, respectively at day 20. The concentrations of uric acid in saliva were 2.8 and 3.7-fold lower in the 9th and 20th day of lactation, respectively, than the first day. Salivary AOPP were 2.1 and 3.4-fold lower at day 9 and 20 of lactation, respectively. The H_2_O_2_ concentrations in the saliva of the sows were 107 and 216-fold lower in the 9th and 20th day of lactation, respectively, than the first day.

## Discussion

To the best of authors’ knowledge, no validation studies of assays for measurement of oxidative stress in saliva of pigs have been performed. We included in the panel of biomarkers three analytes that measure the antioxidant capacity in a global way, such as TEAC, CUPRAC and FRAP. It is recommended to measure these analytes  together since they show different changes depending of the physiopathological situation [18]. We also included an analyte, the acid uric, that in humans is indicated as one of the more appropriate biomarkers to evaluate the antioxidant status in saliva [19]. Finally, two oxidant biomarkers were included: AOPP which is a marker of protein oxidation [20] and H_2_O_2_ which is a product of superoxide anion [21].

The analytical validation tests for assay sensitivity, reproducibility, and accuracy showed that the assays used in our study are valid and reliable for determination of biomarkers of oxidative stress in porcine saliva. They were precise, linear, accurate and reproducible, which is in accordance with previous validation studies with oxidative stress markers in human saliva [22].

All assays evaluated in this study were automated. The automated assays presented here have considerable advantages over the manual ones: set-up time is reduced which makes possible a hundreds of measurements within time periods ranging from minutes to hours, require reduced volume of sample and reagents, operator attendance is not needed once the samples are placed in the automated analyser and operator error is reduced leading to a higher precision and accuracy [23]. Although these assays are adapted to automated analysers, they could be used in other formats such as ELISA plates or in manual spectrophotometric measurements.

It is known that in peripartum, particularly the delivery, and early lactation, energy and consequently oxygen are extremely required. The rapid differentiation of secretory parenchyma, intense mammary gland growth, and the onset of copious milk synthesis and secretion are accompanied by high metabolic demand and elevated requirements for tissue oxygen [24]. This physiological situation can lead to an oxidative stress state due to the overproduction of oxidants [25]. In this regard, there are clinical and experimental evidences that in the periparturient period in sows there is an antioxidant/oxidant imbalance [11, 26]. This lead us to use the lactation time as an experimental model to evaluate the possible changes in analytes of our study in situations of oxidative stress.

Our results showed increased antioxidant and oxidant concentrations in saliva of sows at the first day of lactation which decreased during the 20 days of lactation. It is possible that the overproduction of oxidants and increased response of the antioxidant system have been induced not only by early lactation but also due to farrow. Results of this study were in agreement with a previous report [27] which described increased concentrations of various oxidant compounds in serum of sows such as TBARS, 8-hydroxy-deoxyguanosine (8-OHdG), and ROS during early lactation. These oxidants reached the highest values at first day of lactation and decreased progressively between the third and 21th day of lactation with exception of 8-OHdG that decreased from day 7 and remained with the same concentrations at day 21 [11].

Similar results with increases in antioxidant and oxidant biomarkers in serum at the beginning of lactation were also found in other species. In cows it was reported that plasma FRAP levels were high immediately, and 1 and 2 days after parturition and decreased at 6 and 12 days after delivery [28]. In addition, they indicated an increase in protein oxidation at beginning of lactation. Also a report [29] showed that serum TEAC in cows was higher at beginning of calving when compared with two weeks after.

In our study, the antioxidants and oxidants evaluated had a similar evolution during the 20 days of lactation, which could imply that an increase in antioxidants is produced in order to compensate the overproduction of oxidant compouds that occur during this period. An increase in the antioxidant response in order to reach a balance between antioxidant and oxidant compounds has been described in lactation [28]. Which means that, even a state of oxidative stress occurs in the early lactation, the balance would be tried to be reestablished.

Based on the results of our experimental model, TEAC would be the recommended TAC assay to evaluate changes of antioxidant capacity in saliva of sows in lactation, since showed higher changes than CUPRAC and FRAP between the samples taken at the beginning and end of the lactation, and also showed significant changes between the day 1 of lactation and the other samples times, contrarily to uric acid. Therefore, a profile including TEAC as a biomarker of antioxidant status and H_2_O_2_ and AOPP as oxidant biomarkers might be used for an evaluation of oxidative stress status in saliva of sows during the period of lactation. However, further studies evaluating the behaviour of these biomarkers in saliva samples in different situations of stress or diseases should be made.

Saliva is predominantly composed by watery fluid, however also contains a complex mixture of proteins, ions and other organic compounds originated from salivary glands and blood [30]. Its availability, easy collection and possibility of repeated non-invasive sampling makes it ideal for screening, diagnosis, or monitoring of many diseases [31]. Because of that, salivary markers are very attractive and are becoming a promising tool for the research. In addition, the collection of saliva leads to minimal discomfort to the animal, being considered to be an ideal material for evaluating the stress condition in pigs [32]. This study opens the possibility of use saliva to evaluate the oxidative status in pigs. It would be of interest to perform further studies about how oxidative biomarkers change in saliva in different physiological conditions or situations of stress or diseases, and also to evaluate if those sows with higher values of antioxidant and lower values of oxidant biomarkers in saliva at day 1 of lactation can have a higher performance as described previously in serum [16].

## Conclusions

It can be concluded that the panel of assays for 6 biomarkers of oxidative stress in saliva of pigs that have been evaluated in this study demonstrated a high analytical accuracy and low imprecision, and therefore are valid from an analytical point of view. In addition, it has been observed that these biomarkers can change in a situation of oxidative stress such as lactation in sows. This study opens a new possibility of using saliva as a non-invasive sample to evaluate oxidative stress in pigs.

## Methods

### Spectrophotometric methods

#### TEAC

This assay is based on the enzymatically generation of 2,2′-azino-bis(3-ethylbenzthiazoline-6-sulfonic acid) (ABTS) radical [33]. The assay measures the ability of the non-enzymatic antioxidants present in the sample in reducing the preformed radical. Its disappearance was estimated by decrease in absorbance at 700 nm and Trolox (6-hydroxy-2,5,7,8-tetramethylchroman-2-carboxylic acid), an α-tocopherol analogue, was used as standard. The assays results were expressed in millimoles of Trolox equivalents per liter.

#### CUPRAC

CUPRAC assay is based on the generation of a complex containing Cu^2+^ and one chelating agent, in this case the bathocuproinedisulfonic acid disodium salt, and its reduction to Cu^1+^ by the non-enzymatic antioxidants present in a sample [34]. Results were compared with a standard curve achieved with Trolox and were also expressed in millimoles of Trolox equivalents per liter.

#### FRAP

The FRAP measurement is based on the assay described by Benzie and Strain [37]. A reaction mixture containing ferric-tripyridyltriazine (Fe^3+^) is reduced to the ferrous (Fe^2+^) form by the non-enzymatic antioxidants provided by the sample. Ferric chloride hexahydrate (FeCl_3_·6H_2_) solution was used to produce a standard curve and compare with sample results that were expressed in millimoles of Fe^2+^ equivalents per liter.

#### Uric acid

The uric acid concentrations were measured according to the manufacturer’s instructions of a commercially available spectrophotometric method (Beckman) based on a previously described assay [38].

#### AOPP

Concentrations of AOPP were determined according a previously described method [35]. The assay was calibrated with Chloramine-T solutions that absorb at 340 nm in the presence of potassium iodide in acidic conditions.

#### H_2_O_2_

The H_2_O_2_ assay was based on the method described by Rhee et al. [36]. In this assay, 3,5,3′5′-tetramethylbenzidine (TMB) reacts with horseradish peroxidase (HRP) and H_2_O_2_ and a TMB cation free radical is originated. Further oxidation of this free radical produces the diimine, a yellow-colored oxidation product with maximal absorbance at 450 nm. H_2_O_2_ solution was used to produce a calibration curve and compare with sample results.

### Analytical validation

For the analytical validation of the assay, the following parameters were determined:

#### Reproducibility

Intra-assay precision was determined by the analysis of three saliva samples (one with high, one with medium, and one with low analyte concentration) five times in the same analytical run (same day). Inter-assay precision was determined by the analysis of the same samples on five different days within 1 week. The CV was determined by dividing the SD of the parallel measurements by their mean and then multiplied by 100.

#### Accuracy

Linearity under dilution and spiking recovery were used to assess accuracy. To linearity, two saliva samples were serially diluted with ultrapure water and assayed. The results were compared with those expected by linear regression analysis. To spiking recovery, two saliva samples containing different analyte concentrations were selected and mixed at different percentages. The percentages of recovery were calculated for each dilution as (observed result/expected result) × 100.

#### Assay sensitivity

The assay sensitivity was calculated based on the LOD. It was calculated as 2 times the SD above the mean blank sample value, which was obtained from 20 replicate measurements of the assay buffer or water.

### Biomarkers performance

#### Animals and sampling

The experimental protocol was approved by the Ethical Committee of the Autonomous University of Barcelona (UAB).

A total of 14 multiparous (Landrace × Duroc) sows around farrowing were included in the study. They were subjected to a clinical examination prior to and throughout the study, and no clinical signs of disease were found. All of them were under the same local conditions. They were allocated in farrowing crates with regulated temperature (approximately 20 °C). The lights in the room were on from 7 am to 5 pm. The sows ate the same diet at 7 am and 3 pm (a total of 2.6 kg) and had water ad libitum. These sows were from a commercial farm (l’Heura S.L., Santa Perpetua de Mogoda, Barcelona, Spain) and were maintained in their productive system after the experiment.

The sows were induced to farrow by using Cloprostenol 0.092 mg/ml (1 ml at 7 am and other at 11 am) at day 113 of gestation. Treatments and manual interventions during farrowing were performed by the same person following the usual routine of the farm.

Saliva samples were taken at day 1, 9 and 20 of lactation using Salivette® tubes (Sarstedt AG& Co., Germany), containing a cotton swab which was clipped through a Kocher clip, and was kept in the sows’ mouth during 1–2 min. The cotton swab was then placed in the tube and centrifuged at 6000 rpm for 13 min. Finally, saliva samples were stored in Eppendorf tubes and frozen at − 80° until analysis.

### Statistical analysis

Analysis were performed using routine descriptive statistical procedures and commercial software tool (Excel 2016, Microsoft; GraphPad Prism 6). D’Agostino& Pearson omnibus normality test was performed to assess normality of data. In the trial in which analytes were evaluated in lactation, Friedman test followed by the Dunn’s multiple comparison test was used to evaluate differences in analytes between the different sampling time-points except for CUPRAC that statistical significance was determined by ANOVA once a parametric distribution was given. For all tests, *P* < 0.05 was considered as statistically significant.

## Abbreviations

AOPP: advanced oxidation protein products
CUPRAC: cupric reducing antioxidant capacity
FRAP: ferric reducing ability of plasma
H_2_O_2_: hydrogen peroxide
LOD: limit of detection
TAC: total antioxidant capacity
TEAC: trolox equivalent antioxidant capacity
